# Effect of solid-state fermentation and ultrasonication processes on antimicrobial and antioxidant properties of algae extracts

**DOI:** 10.3389/fnut.2022.990274

**Published:** 2022-08-26

**Authors:** Ernesta Tolpeznikaite, Vytaute Starkute, Egle Zokaityte, Modestas Ruzauskas, Renata Pilkaityte, Pranas Viskelis, Dalia Urbonaviciene, Romas Ruibys, João M. Rocha, Elena Bartkiene

**Affiliations:** ^1^Institute of Animal Rearing Technologies, Faculty of Animal Sciences, Lithuanian University of Health Sciences, Kaunas, Lithuania; ^2^Department of Food Safety and Quality, Faculty of Veterinary, Lithuanian University of Health Sciences, Kaunas, Lithuania; ^3^Department of Anatomy and Physiology, Faculty of Veterinary, Lithuanian University of Health Sciences, Kaunas, Lithuania; ^4^Institute of Microbiology and Virology, Faculty of Veterinary, Lithuanian University of Health Sciences, Kaunas, Lithuania; ^5^Marine Research Institute, Klaipeda University, Klaipeda, Lithuania; ^6^Lithuanian Research Centre for Agriculture and Forestry, Institute of Horticulture, Babtai, Lithuania; ^7^Institute of Agricultural and Food Sciences, Vytautas Magnus University, Agriculture Academy, Kaunas, Lithuania; ^8^Laboratory for Process Engineering, Environment, Biotechnology and Energy, Faculty of Engineering, University of Porto, Porto, Portugal; ^9^Associate Laboratory in Chemical Engineering, Faculty of Engineering, University of Porto, Porto, Portugal

**Keywords:** solid-state fermentation, ultrasonication, algae, extracts, antimicrobial properties, antioxidant properties, lactic acid bacteria

## Abstract

Algal biomass (AB) is prospective source of valuable compounds, however, Baltic Sea macroalgae have some challenges, because of their high microbial and chemical contamination. These problems can be solved, by using appropriate technologies for AG pre-treatment. The aim of this study was to evaluate the influence of two pre-treatments, solid-state fermentation with the *Lactiplantibacillus plantarum* LUHS135 and ultrasonication, on the antioxidant and antimicrobial characteristics of macro- (*Cladophora rupestris, Cladophora glomerata, Furcellaria lumbricalis, Ulva intestinalis*) and Spirulina (*Arthrospira platensis*) extracts. Also, combinations of extracts and LUHS135 were developed and their characteristics were evaluated. The total phenolic compound content was determined from the calibration curve and expressed in mg of gallic acid equivalents; antioxidant activity was measured by a Trolox equivalent antioxidant capacity assay using the DPPH^•^ (1,1-diphenyl-2-picrylhydrazyl), ABTS^•+^ 2,2′-azinobis-(3-ethylbenzothiazoline-6-sulfonic acid), FRAP (Ferric Reducing Ability of Plasma) discoloration methods. Antimicrobial activity was measured by using agar well diffusion assay and in a liquid medium. The highest DPPH^•^ and ABTS^•+^ was shown by *C.rupestris* and *F.lumbricalis* extract × LUHS135 combinations, the highest FRAP - by non-pretreated *C.rupestris* and *F.lumbricalis* extract × LUHS135 combinations. Ultrasonicated samples inhibited four out of seven tested pathogens. Finally, the tested pre-treatments showed good perspectives and can be recommended for AB valorization.

## Introduction

Algal biomass can be converted into a wide range of functional products (1). Despite, that they are a valuable source of functional compounds, our previous studies showed that the application of Baltic Sea macroalgae have some challenges because of their high microbial and chemical contamination (2). However, algae safety parameters could be improved by applying ethanolic extraction, which is a suitable technology for pathogen decontamination and reduces the toxic metal concentration in algae extracts (3). In addition to improvements in algae products' safety parameters, it would be very beneficial to increase extraction efficiency. Therefore, in this study, two methods for algae pre-treatment were tested before extraction: (I) solid-state fermentation (SSF) with a selected lactic acid bacteria (LAB) strain and (II) ultrasonication. We hypothesized that algae biomass pre-treatment before extraction can lead to better properties of the extracts (higher antioxidant activity and total phenolic compound (TPC) content, as well as stronger antimicrobial properties against a broader spectrum of pathogenic and opportunistic strains). In addition, to increase the antimicrobial and antioxidant activity of the prepared extracts, combinations of algae extracts and a pure *Lactiplantibacillus plantarum* LUHS135 strain were developed. Our previous studies showed that the above-mentioned strain inhibits various pathogenic and opportunistic microorganisms and is suitable for fermentation of various substrates (4–7). The importance of algae biomass pre-treatment before extraction can be explained by algae cell composition, which is protected by complex cell walls (8, 9). It has been reported that the crucial step in obtaining bioactive compounds from micro- and macroalgal biomass is to achieve efficient cell disruption (10). Some algae pre-treatment technologies are described in the literature, and the most effective mechanical and biological techniques were mentioned (11, 12). Despite the fact that physical pre-treatment was found to be a cost-intensive process, ultrasonication was recommended as the most promising method for cell disintegration (9, 13, 14). Ultrasound breaks the cell structure and improves material transfer by enhancing the extraction from microalgae (9, 15–17). Also, biological pre-treatment with fungi, bacteria and/or their enzymes can be used to degrade lignin and hemicelluloses of algae cells (12, 18). There are numerous studies on algae pre-treatment using biological tools (19–21). In addition to the breakdown of lignin, biological pre-treatment generates other valuable compounds such as phenolic acids, benzoic acid, syringaldehyde, etc. (22). Other major advantages of biological pre-treatment are low energy consumption, simple operating conditions and equipment, no requirement for recycling the chemicals after pre-treatment, etc. (23–25). Solid state fermentation (SSF) process is based on the microorganisms grown on solid or semi-solid substrates or supports, and is more effective than the liquid phase submerged fermentation (26). We hypothesized that algae biomass SSF can lead to the deeper algae cells breakdown, which will lead to better properties of the extracts.

The aim of this study was to evaluate the influence of two pre-treatments, solid-state fermentation (SSF) with the *Lactiplantibacillus plantarum* LUHS135 strain and ultrasonication (for 45 min at 35 kHz), on the antioxidant and antimicrobial characteristics of macroalgae (*Cladophora rupestris, Cladophora glomerata, Furcellaria lumbricalis* and *Ulva intestinalis*) and microalgae [Spirulina (*Arthrospira platensis*)] extracts. In addition, combinations of algae extracts and the pure LUHS135 strain were developed and their antioxidant and antimicrobial characteristics were evaluated.

## Materials and methods

### Algae samples and lactic acid bacteria strain used in experiments

Samples of macroalgae (*Furcellaria lumbricalis, Ulva intestinalis, Cladophora rupestris* and *Cladophora glomerata*) were collected in May–June of 2021 on the Lithuanian coast. *Ulva intestinalis* and *C. glomerata* samples were taken from stones near the surface, while *F. lumbricalis* and *C. rupestris* samples were taken after a storm along the shore. The collected samples were cleaned three times in distilled water to remove sand and macroscopic invertebrates. Microalgae Spirulina (*Arthrospira platensis*) was purchased from the University of Texas Biological Labs (Austin, Texas, United States), multiplied according to instructions given by producer and used in experiments.

Before the experiments, all algal samples were lyophilized using a freeze-dryer FD8512S (ilShin® Europe, Ede, The Netherlands) and ground into a powder (particle size <0.2 mm) using a knife mill GM200 (Retsch, Düsseldorf, Germany). Freeze-dried samples were maintained at room temperature in a dark place until they were used.

The *Lactiplantibacillus plantarum* LUHS135 strain (LUHS135) was obtained from the Lithuanian University of Health Sciences collection (Kaunas, Lithuania). The characteristics of the LAB strain used, including the inhibition of strains of pathogenic and opportunistic bacteria, and fungi are described by Bartkiene et al. (4). In addition, our previous studies showed that fermentation of feed with LUHS135 had a positive influence *in vivo* on piglets' health parameters (27–29). The above-mentioned LAB strains were stored at−80°C in a Microbank system (Pro-Lab Diagnostics, United Kingdom) and propagated in de Man–Rogosa–Sharpe (MRS) broth (CM 0359, Oxoid Ltd, Hampshire, United Kingdom) at 30 ± 3°C for 48 h before their use for algae fermentation.

### Fermentation and ultrasonication of algal samples

The LUHS135 strain was multiplied as described in Algae samples and lactic acid bacteria strain used in experiments and used for algae powder (AP) fermentation. A total of 3 mL of the LAB strain multiplied in MRS (cell concentration, on average, 9.0 log_10_ CFU mL^−1^) was inoculated to 100 g of AP media (for 100 g of AP, 60 mL of water was used) and fermented at 30 ± 2°C for 60 h. Control samples for pH analysis were prepared without the addition of LAB. Our previous studies showed that pure algae samples are not suitable substrates for effective LAB growth (2), thus 2% (from the algae sample amount) of yeast extract was added (ThermoFisher, Kandel, Germany), with the purpose of improving the growth of the LUHS135. Anaerobic conditions were attained by incubating the fermentable substrate in anaerobic jars (Oxoid, Basingstoke, Hampshire, United Kingdom), with GasPak Plus™ (BBL, Cockeysville MD, United States). Samples for pH analysis were taken after 12, 24, 36, 48 and 60 h of fermentation.

Algae samples were ultrasonicated before extract preparation for 45 min at 35 kHz (temperature of samples during the ultrasonication was 40 ± 2°C) using ultrasonic bath (Bandelin Sonorex, Bandelin electronic GmbH & Co. KG, Berlin, Germany).

Both fermented and ultrasonicated algae samples were lyophilised and used for extract preparation.

### Extracts and extract × l*actiplantibacillus plantarum* LUHS135 strain combinations preparation

Five grams of the lyophilized algal samples (non-pretreated, fermented and ultrasonicated, for a total of 15 samples) were extracted with 100 mL of ethanol/water (70:30 v/v) (30) by incubation at room temperature (22 ± 2°C) overnight with stirring (Vibramax 100, Heidolph, Nuremberg, Germany). Then, extracts were centrifuged at 3,500 rpm for 10 min at 4°C and filtered through Whatman No. 4 filter paper. Ethanol was removed by rotary evaporation in the extract. The concentrate and the supernatant of the extract were lyophilized and weighted.

**Figure 1:**
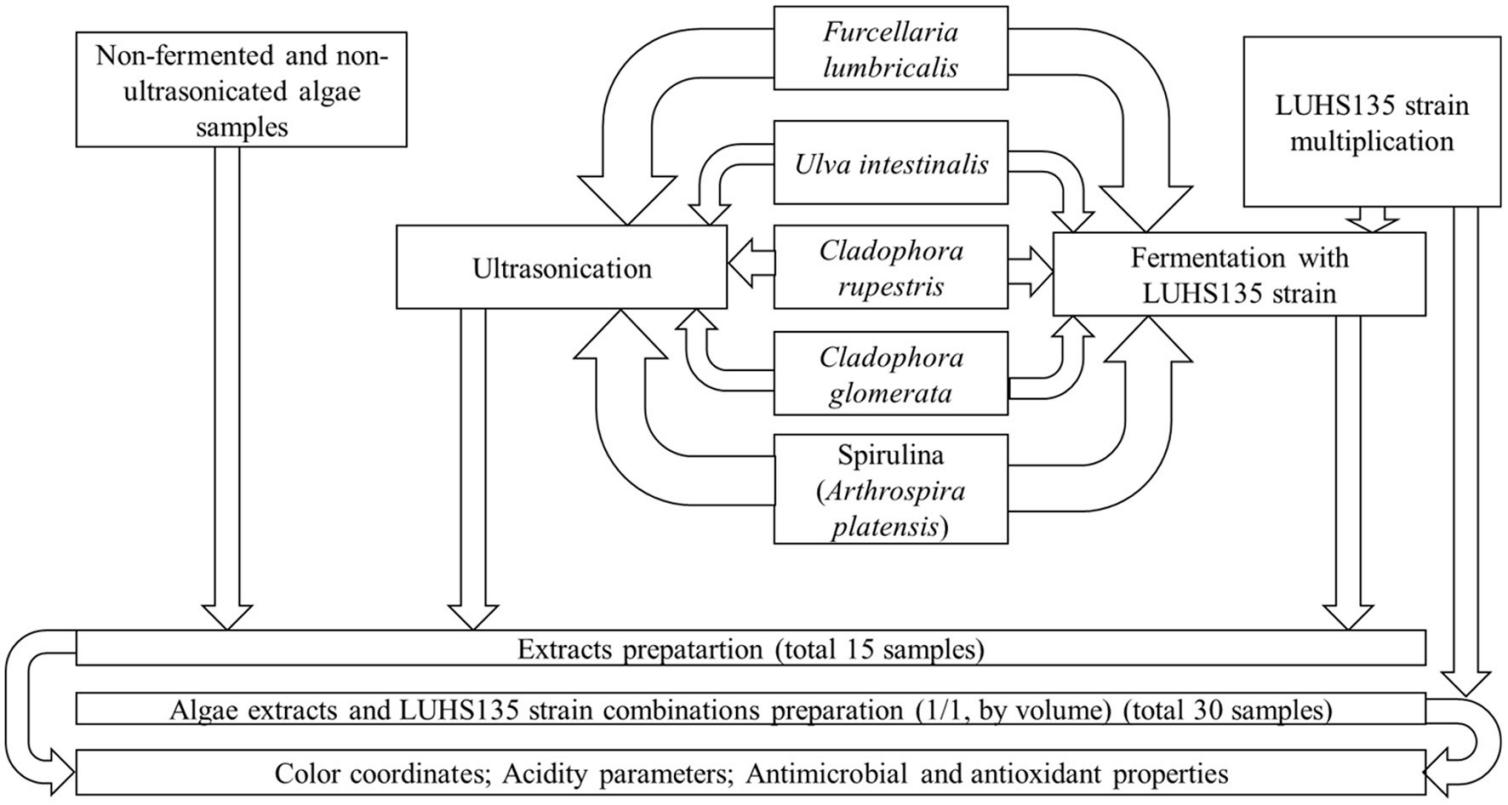
The principal scheme of the experiment.

For the preparation of extract × LUHS135 strain combinations, it was propagated in MRS broth (CM 0359, Oxoid Ltd, Hampshire, United Kingdom) at 30 ± 3°C for 48 h, and a pure LUHS135 strain was used (LUHS135 strain/algae extract; 50/50, by volume). The principal scheme of the experiment is given in Figure 1. Three groups of samples were prepared: (I) extracts and extracts × LUHS135 combinations prepared from non-pre-treated algae, (II) extracts and extracts × LUHS135 combinations prepared from ultrasonicated algae and (III) extracts and extracts × LUHS135 combinations prepared from fermented algae. In every group pure extract as well as extract combinations with the LUHS strain were tested (ClaR = *Cladophora rupestris*; ClaG = *Cladophora glomerata*; Ul = *Ulva intestinalis*; Furc = *Furcellaria lumbricalis*; Sp = Spirulina (*Arthrospira platensis*); non = extracts prepared from non-pre-treated algae; ultr = extracts prepared from ultrasonicated algae; ferm = extracts prepared from fermented algae; LUHS135 = extract × LUHS135 strain combination). There were 30 samples total: Group (I): ClaR_non_, ClaR_nonLUHS135_, ClaG_non_, ClaG_nonLUHS135_, Furc_non_, Furc_nonLUHS135_, Ul_non_, Ul _nonLUHS135_, Sp_non_ and Sp_nonLUHS135;_ Group (II): ClaR_ultr_, ClaR_ultrLUHS135_, ClaG_ultr_, ClaG_ultrLUHS135_, Furc_ultr_, Furc_ultrLUHS135_, Ul_ultr_, Ul_ultrLUHS135_, Sp_ultr_ and Sp_ultrLUHS135_ and Group (III): ClaR_ferm_, ClaR_fermLUHS135_, ClaG_ferm_, ClaG_fermLUHS135_, Furc_ferm_, Furc_fermLUHS135_, Ul_ferm_, Ul_fermLUHS135_, Sp_ferm_ and Sp_fermLUHS135_.

### Analysis of algae color characteristics and pH

The color coordinates of the algae extracts and their combinations with the LUHS135 strain were evaluated using a CIE L^*^a^*^b^*^ system (CromaMeter CR-400, Konica Minolta, Marunouchi, Tokyo, Japan) (3). The pH of samples was evaluated with an “inoLab pH Level 3” pH meter (Hanna Instruments, Weilheim, Germany).

### Determination of the total phenolic compound content

The total phenolic compound (TPC) content in the extracts was determined according to the Folin–Ciocalteu method (31) with slight modifications (32). Samples (1.0 mL) were introduced into test cuvettes followed by 5.0 mL 10% (1/10, v/v) of Folin–Ciocalteu's reagent by diluting a stock solution with ultra-pure distilled water and 4.0 mL of Na_2_CO_3_ (7.5%). The system was then placed at ambient temperature for 1 h. The absorbance was measured at 765 nm using a Genesys-10 UV/VIS spectrophotometer (Thermo Spectronic, Rochester, NY, United States). The concentration of TPC was determined from the calibration curve and expressed in mg of gallic acid equivalents (GAE) in ml of extracts.

### Determination of the antioxidant capacity of algae extracts

The antioxidant activity of algae extracts was measured by DPPH^•^, ABTS^•+^ and FRAP discoloration methods. Calculation of all antioxidant activity assays was carried out using Trolox calibration curves and expressed as μmol of the Trolox equivalent (TE) per one gram of ml of extract (μmol TE/ml).

#### DPPH^•^ activity

The DPPH^•^ (2,2-diphenyl-1-picrylhydrazyl hydrate free radical) scavenging capacity of the algal extracts was determined by the method of Brand-Williams et al. (33) with slight modifications (34). Twenty microliters of extract were allowed to react with 2 mL of DPPH^•^ ethanolic solution (2 mL, 6 × 10^−5^ M) by mixing in a cuvette with a 1 cm path length for 30 min in the dark. The decrease in absorbance was measured at 515 nm using a Genesys-10 UV/VIS spectrophotometer (Thermo Spectronic, Rochester, NY, United States).

#### ABTS^•+^ activity

The radical scavenging activity of extracts was also measured by ABTS^•+^ (2,2′-azino-bis(3-ethylbenzthiazoline-6-sulphonic acid) radical cation assay (35) as described by Urbonaviciene et al. (32). ABTS^•+^ solution (2 mM) was prepared by dissolving 2,2′-azinobis (3-ethylbenzothiazoline-6-sulfonic acid) diammonium salt in 50 mL of phosphate-buffered saline (PBS) obtained by dissolving 8.18 g NaCl, 0.27 g KH_2_PO_4_, 1.42 g Na_2_HPO_4_ and 0.15 g KCl in 1 L of pure water. The pH of the prepared solution was adjusted to 7.4 using NaOH. Then the K_2_S_2_O_8_ solution (70 mM) was prepared in pure water. Briefly, 2 mL of ABTS^•+^ radical solution was mixed with 20 μL extract also in a 1 cm path length cuvette. The reaction mixture was kept at ambient temperature in the dark for 30 min, and the absorbance was read at 734 nm using a Genesys-10 UV/Vis spectrophotometer (Thermo Spectronic, Rochester, NY, United States). Trolox was used as a standard. A duplicate determination was made from each extract.

#### FRAP activity

The ferric reducing antioxidant power (FRAP) assay was carried out by the method of Benzie and Strain (36) with some modifications (37). For the FRAP assay, 0.3 M of sodium acetate buffer (pH 3.6) was prepared by dissolving 3.1 g of sodium acetate and 16 mL of acetic acid in 1,000 mL of distilled water; a 10 mM TPTZ solution was prepared by dissolving 0.031 g of TPTZ in 10 mL of 40 mM HCl; and a 20 mM ferric solution was prepared by dissolving 0.054 g of FeCl_3_·6H_2_O in 10 mL of distilled water. Working FRAP reagent was prepared by freshly mixing acetate buffer, TPTZ and ferric solutions at a ratio of 10:1:1. Two milliliters of freshly prepared FRAP working solution and 20 μL of extract were mixed and incubated for 30 min at ambient temperature. The change in absorbance due to the reduction of the ferric-tripyridyltriazine (Fe III-TPTZ) complex by the antioxidants present in the samples was measured at 593 nm using a Genesys-10 UV/VIS spectrophotometer.

### Evaluation of the antimicrobial activity of algal extract samples

The algal extracts as well as algal extract × LUHS135 strain combination antimicrobial properties were evaluated by testing their abilities to inhibit the following pathogenic and opportunistic strains: *Salmonella enterica, Bacillus cereus, Enterococcus faecium, Staphylococcus aureus, Escherichia coli, Streptococcus mutans* and *Enterococcus faecalis*. Antimicrobial properties of the samples were evaluated by using the agar well diffusion method and in a liquid medium.

For the agar well diffusion assay, suspensions of 0.5 McFarland standard of each pathogenic bacterial strain were inoculated onto the surface of cooled Mueller–Hinton agar (Oxoid, Basingstoke, UK) using sterile cotton swabs. Wells 6 mm in diameter were punched in the agar and filled with 50 μL of the algal extract. The antimicrobial activities against the tested bacteria were established by measuring the inhibition zone diameters (mm). The experiments were repeated three times, and the average diameter of the inhibition zones in mm was calculated.

To evaluate the antimicrobial activity of the algal extracts and algal extracts × LUHS135 combinations in liquid medium, the algal samples were diluted 1:3 (v/v) with physiological solution. Then we added 10 μL of the pathogenic and opportunistic bacterial strains, cultured in a selective medium, to the different concentrations of samples (500 and 2,000 μL) and incubated them at 35°C for 24 h. After incubation, the viable pathogenic and opportunistic bacterial strains in algal extract and/or in algal extracts × LUHS135 combination were controlled by plating them on selective medium. The results were interpreted as (–) if the pathogens did not grow on the selective medium and (+) if the pathogens grew on the selective medium. Experiments were performed in triplicate.

### Statistical analysis

Extract preparation of algal samples was performed in duplicate, while all analytical experiments were carried out in triplicate. The calculated mean values, using the statistical package SPSS for Windows (Ver.15.0, SPSS, Chicago, IL, United States), were compared using Duncan's multiple range test with significance defined at *p* ≤ 0.05. A linear Pearson's correlation was used to quantify the strength of the relationship between the variables. The results were recognized as statistically significant at *p* ≤ 0.05.

## Results and Discussion

### Selection of algae fermentation duration before extract preparation according to changes in their pH

**Figure 2:**
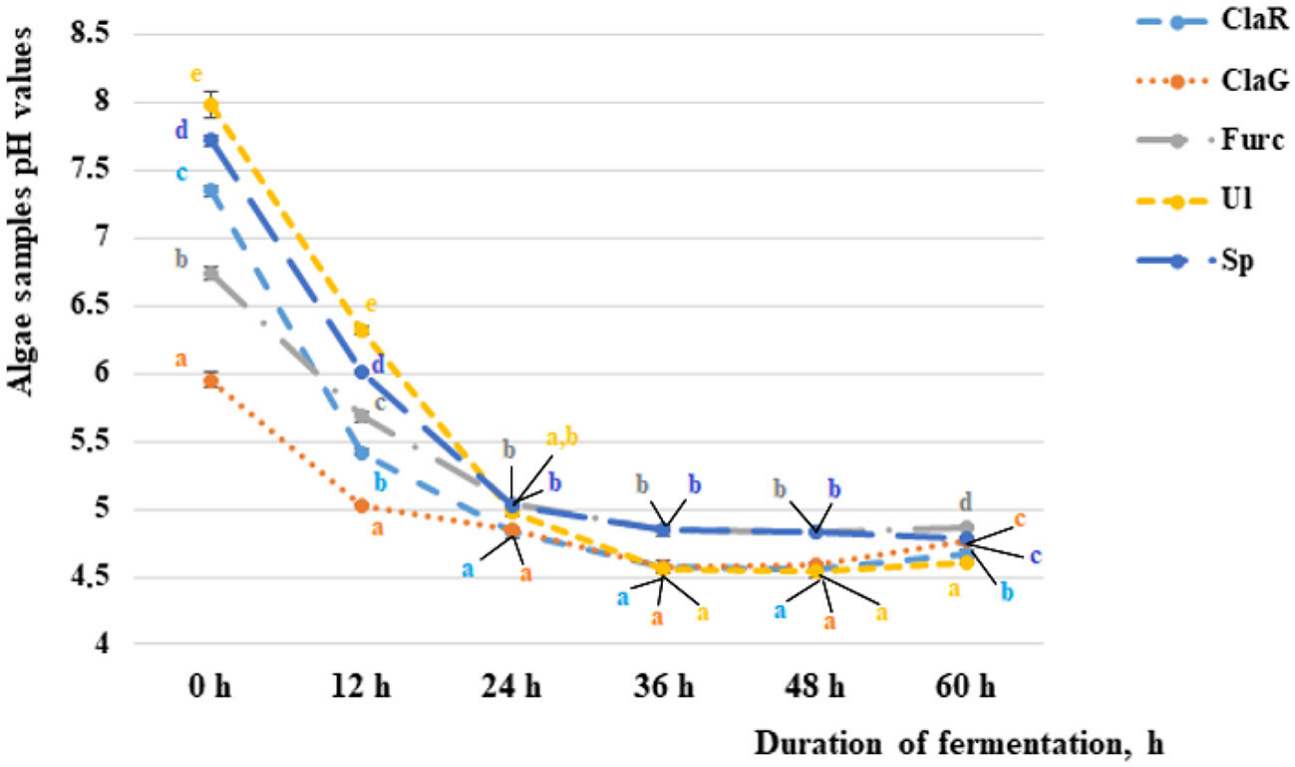
The pH of the non-fermented and fermented algae samples after 12, 24, 36, and 60 h of fermentation [ClaR - *Cladophora rupestris*; ClaG - *Cladophora glomerata*; Ul - *Ulva intestinalis*; Furc - *Furcellari lumbricalis*; Sp - Spirulina (*Arthrospira platensis*)]. Data are represented as means (*n* = 3) ± SE. Means with different letters (a–e) are significantly different (*p* ≤ 0.05). The color of the letters coincides with the color of the sample in the graph.

The changes in pH values during algae fermentation are shown in Figure 2. In comparison to the non-fermented samples, a pH higher than 7.0 was established for *Cladophora rupestris, Ulva intestinalis* and Spirulina samples (7.35, 7.98 and 7.72, respectively). Non-fermented *Cladophora glomerata* and *Furcellaria lumbricalis* samples had average pH values of 5.95 and 6.74, respectively. The most intensive fermentation and reductions of pH values was found from 0–12 h and from 12–24 h of fermentation. From 0–12 h and from 12–24 h of fermentation the pH values of *Cladophora rupestris, Cladophora glomerata, Ulva intestinalis, Furcellaria lumbricalis* and Spirulina samples reduced by an average of 1.36 and 1.12, 1.18 and 1.04, 1.17 and 1.13, 1.26 and 1.27 and 1.28 and 1.19 times, respectively.

Although fermentation during the period from 24–36 h was not as intensive as fermentation in previous studies, after 36 h of fermentation significantly lower pH values for all of the tested algae samples were found when compared with samples fermented for 24 h. However, after 48 h of fermentation significant differences between the algae pH values were not found, and after 72 h of fermentation some of the algae sample pH values started to increase. Univariate analyses of variance showed that the variety of algae is a significant factor in sample pH (*p* = 0.017). However, the duration of fermentation and interaction with analyzed factors did not significantly affect the pH of the samples. According to these results, a fermentation duration of 36 h for extract preparation was selected.

Literature on algae fermentation is scarce; however, our previous studies showed that fermentation of the LUHS135 strain (duration of fermentation 12 h) significantly reduced the pH of *C. rupestris*. However, the pH of other tested algae samples (*U. intestinalis* and *F. lumbricalis*) remained unchanged (2). One of the main goals of the fermentation process is to drop the pH, and on average, the recommended pH for fermented food is 4.6. A decrease in pH is an indicator of an effective process; however, changes to the fermentable substrate can be caused by many factors, i.e., the technological microorganism's (used for fermentation) characteristics, nutrient source in fermentable media, duration of fermentation, humidity of the substrate, etc. It has been reported that the moisture content of the substrate has a significant influence on pH and, in most cases, lower pH values and higher total titratable acidity were obtained for peas in solid state fermentation conditions (38). The practice of LAB-based food, as well as feed fermentations, happened accidentally in the beginning, but soon spread due to its many benefits including nutrition, safety and flavor (38, 39). Overall, during the fermentation process many compounds are obtained as secondary metabolites of technological microorganisms (40, 41). Also, bound phenolic compounds are bio-converted from their conjugated forms to their free forms, and this is explained by their breakdown, activities of the fermentable substrate enzymes, as well as activity of technological microorganisms (42). Finally, this study showed that yeast extract is a suitable supplement for increasing algae samples fermentation effectiveness.

### Color coordinates and pH of algae extracts and algae extracts × LUHS135 combinations

Color coordinates (L^*^ = lightness; a^*^ = redness; -a^*^ = greenness; b^*^ = yellowness; -b^*^ = blueness) and pH of the algae extracts and algae extracts × LUHS135 combinations are shown in Table 1. When comparing all three groups of extracts (non-pre-treated, ultrasonicated and fermented before extraction), the lowest L^*^ coordinates were from ClaG_non_, ClaR_ultr_ and ClaR_fermLUHS135_ samples (42.5, 41.3 and 49.5 NBS, respectively). The most intensive greenness (-a^*^) was from Ul_non_, Ul_ultr_ and Ul_ferm_ samples (-14.7,−13.7 and−6.86 NBS, respectively). The lowest yellowness (b^*^) was from ClaG_non_, Sp_ultr_ and ClaG_ferm_ samples (24.8, 23.7 and 23.1 NBS, respectively).

**Table 1:** Color coordinates (L^*^, lightness; a^*^, redness; –a^*^, greenness; b^*^, yellowness; –b^*^, blueness) and pH of the algae extracts and algae extracts × LUHS135 combinations.

**Extracts and extract × LUHS135 combination**	**Color coordinates, NBS**			**pH**	**Multivariate analysis of variance**		
	L*	a*	b*		**Factor**	**Depen-dent variable**	***p***
Extracts and extracts × LUHS135 combinations prepared from non-pre-treated algae					Algae species	L*	0.0001
ClaR_non_	64.6 ± 0.32^g^	−13.8 ± 0.11^b^	47.5 ± 0.36^g^	6.77 ± 0.031^d^		a*	0.0001
ClaR_nonLUHS135_	61.1 ± 0.26^e^	−1.40 ± 0.15^g^	44.6 ± 0.33^e^	3.95 ± 0.032^a^		b*	0.0001
ClaG_non_	42.5 ± 0.10^a^	−1.75 ± 0.192^f^	24.8 ± 0.18^a^	5.92 ± 0.124^b^		pH	0.712
ClaG_nonLUHS135_	50.6 ± 0.12^b^	2.61 ± 0.105^h^	34.8 ± 0.39^c^	3.96 ± 0.115^a^	Pretreatment used before	L*	0.0001
Furc_non_	79.2 ± 0.34^h^	−3.57 ± 0.022^c^	32.2 ± 0.16^b^	6.19 ± 0.036^c^	extracts preparation	a*	0.0001
Furc_nonLUHS135_	60.5 ± 0.25^d^	10.4 ± 0.24^k^	47.8 ± 0.25^g^	3.92 ±0 .025^a^		b*	0.0001
Ul_non_	52.4 ± 0.32^c^	−14.7 ± 0.16^a^	41.3 ± 0.37^d^	6.99 ± 0.092^e^		pH	0.052
Ul _nonLUHS135_	62.9 ± 0.13^f^	−2.27 ± 0.031^e^	45.8 ± 0.33^f^	3.95 ± 0.071^a^	Extract × LUHS135	L*	0.0001
Sp_non_	59.9 ± 0.32^d^	−3.40 ± 0.114^d^	49.1 ± 0.31^h^	8.69 ± 0.102^f^	combination interaction	a*	0.0001
Sp_nonLUHS135_	64.6 ± 0.10^g^	4.04 ± 0.015^j^	44.9 ± 0.12^e^	3.94 ± 0.044^a^		b*	0.0001
Extracts and extracts × LUHS135 combinations prepared from ultrasonicated algae						pH	0.0001
ClaR_ultr_	41.3 ± 0.31^a^	−1.55 ± 0.064^d^	24.4 ± 0.21^b^	5.82 ± 0.032^b^	Algae species ×	L*	0.0001
ClaR_ultrLUHS135_	45.0 ± 0.24^b^	3.42 ± 0.121^j^	29.2 ± 0.10^c^	3.94 ± 0.091^a^	pre-treatment interaction	a*	0.0001
ClaG_ultr_	50.8 ± 0.37^c^	−7.16 ± 0.092^b^	33.5 ± 0.34^d^	6.37 ± 0.034^d^		b*	0.0001
ClaG_ultrLUHS135_	59.8 ± 0.36^f^	−0.65 ± 0.021^f^	40.0 ± 0.32^e^	3.93 ± 0.022^a^		pH	0.058
Furc_ultr_	71.8 ± 0.44^h^	2.23 ± 0.105^g^	52.6 ± 0.35^j^	6.09 ± 0.093^c^	Algae species × LUHS135	L*	0.0001
Furc_ultrLUHS135_	65.1 ± 0.26^g^	4.43 ± 0.113^k^	45.7 ± 0.22^g^	3.89 ± 0.031^a^	combination interaction	a*	0.0001
Ul_ultr_	55.4 ± 0.37^d^	−13.7 ± 0.24^a^	45.7 ± 0.34^g^	7.01 ± 0.074^e^		b*	0.0001
Ul_ultrLUHS135_	57.1 ± 0.10^e^	−1.26 ± 0.031^e^	47.1 ± 0.12^h^	3.92 ± 0.032^a^		pH	0.362
Sp_ultr_	79.9 ± 0.41^j^	−5.40 ± 0.154^c^	23.7 ± 0.24^a^	7.67 ± 0.107^f^	Pre-treatment × LUHS135	L*	0.0001
Sp_ultrLUHS135_	65.3 ± 0.31^g^	5.17 ± 0.072^l^	44.6 ± 0.27^f^	3.92 ± 0.094^a^	combination interaction	a*	0.0001
Extracts and extracts × LUHS135 combinations prepared from fermented algae						b*	0.0001
ClaR_ferm_	54.7 ± 0.25^b^	−4.55 ± 0.094^b^	33.5 ± 0.34^c^	5.09 ± 0.064^b^		pH	0.031
ClaR_fermLUHS135_	49.5 ± 0.37^a^	3.33 ± 0.046^f^	34.5 ± 0.22^d^	4.02 ± 0.084^a^	Algae species ×	L*	0.0001
ClaG_ferm_	63.2 ± 0.22^e^	1.95 ± 0.164^d^	23.1 ± 0.40^a^	5.06 ± 0.040^b^	pre-treatment × LUHS135	a*	0.0001
ClaG_fermLUHS135_	62.4 ± 0.24^d^	7.75 ± 0.140^h^	45.8 ± 0.41^g^	4.07 ± 0.011^a^	combination interaction	b*	0.0001
Furc_ferm_	65.6 ± 0.27^g^	4.67 ± 0.021^g^	43.8 ± 0.44^f^	5.59 ± 0.064^c^		pH	0.004
Furc_fermLUHS135_	64.0 ± 0.38^f^	8.31 ± 0.163^j^	48.0 ± 0.31^h^	4.06 ± 0.052^a^			
Ul_ferm_	76.8 ± 0.25^j^	−6.86 ± 0.111^a^	31.6 ± 0.22^b^	4.95 ± 0.081^b^			
Ul_fermLUHS135_	56.8 ± 0.42^c^	6.50 ± 0.202^h^	41.7 ± 0.14^e^	3.97 ± 0.094^a^			
Sp_ferm_	83.1 ± 0.14^k^	−1.67 ± 0.174^c^	31.5 ± 0.15^b^	5.20 ± 0.107^b^			
Sp_fermLUHS135_	71.7 ± 0.21^h^	3.08 ± 0.037^e^	43.2 ± 0.38^f^	3.98 ± 0.075^a^			

ClaR, Cladophora rupestris; ClaG, Cladophora glomerata; Ul, Ulva intestinalis; Furc, Furcellaria lumbricalis; Sp, Spirulina (Arthrospira platensis); non, extracts prepared from non-pre-treated algae; ultr, extracts prepared from ultrasonicated algae; ferm, extracts prepared from fermented algae; LUHS135, extract × LUHS135 strain combination; L*, ightness; a*, redness; –a^*^, greenness; b*, yellowness; –b*, blueness; NBS, National Bureau of Standards units; data are represented as means (*n* = 3 replicates of analysis) ± SE. a–l indicate the same analytical parameters in different algae species groups. Means with different letters are significantly different (p ≤ 0.05).

When comparing all of the samples, all of the analyzed factors as well as their interactions had significant effects on all color coordinates; however, algae species, pre-treatment used before extract preparation, extract × LUHS135 combination interaction, algae species × pre-treatment interaction and the algae species × LUHS135 combination interaction did not have significant effects on pH of samples (Table 1). In contrast, the pre-treatment × LUHS135 combination interaction, as well as the algae species × pre-treatment × LUHS135 combination interaction, showed a significant influence on sample acidity (*p* = 0.031 and *p* = 0.004, respectively). Also, a weak, negative correlation between the sample pH and a^*^ coordinate was found (r = −0.289, *p* = 0.006) (Table 2). In all cases, the addition of the LUHS135 multiplied strain reduces the algae extracts × LUHS135 combinations until an average pH of 3.96; however, the highest pH was for Sp_non_, Sp_ultr_ and Furc_ferm_ samples (8.69, 7.67 and 5.59, respectively).

**Table 2:** Correlations between the color coordinates (L^*^ = lightness; a^*^ = redness; –a^*^ = greenness; b^*^ = yellowness; –b^*^ = blueness) and pH of the algae extracts and algae extracts × LUHS135 combinations.

**Parameters**	**Pearson correlation (r) and significance (*p*)**	**Parameters**			
		L*	a*	b*	pH
L*	r	1	−0.157	0.452**	0.135
	p		0.140	0.0001	0.205
a*	r	−0.157	1	−0.065	−0.289**
	p	0.140		0.546	0.006
b*	r	0.452**	−0.065	1	0.093
	p	0.0001	0.546		0.386
pH	r	0.135	−0.289**	0.093	1
	p	0.205	0.006	0.386	

^**^Correlation (r) is significant (p) at the 0.01 level (2-tailed).

The color changes can be explained by the fact that during fermentation, the substrate is acidified, and organic acids have an influence on oxidation processes which can lead to color changes (38). In many cases, colored compounds lead to higher antioxidant properties of the product and/or extract; however, specific antioxidant properties are related to specific phenolic compound profile composition (3). However, oxidation of diffused phenolic compounds can also occur (43). In addition to fermentation, ultrasonication could cause color changes in compounds. Ultrasonic waves cause rapid compressions and expansions and destroy substrate cells, and the phenomenon of cavitation is responsible for a reduction of the diffusion boundary layer (44–48). It has been reported that ultrasonication increases extraction efficiency (49, 50). However, other published studies showed that the use of ultrasound as a pre-treatment for carrots contributed to significant changes in their color (51). From this point of view, it is very important to evaluate the changes of the antioxidant properties of the treated samples because reductions in colored compounds could lead to lower antioxidant activity. For this reason, during the second stage of the experiment, antioxidant activities and total phenolic compound content were analyzed.

### The total phenolic compound and antioxidant activity of algae extracts and algae extracts × LUHS135 combinations

The aim of this study was to evaluate Also, combinations of extracts and LUHS135 were developed and their characteristics were evaluated. The total phenolic compounds content was determined from the calibration curve and expressed in mg of gallic acid equivalents; antioxidant activity was measured by a Trolox equivalent antioxidant capacity assay using the DPPH^•^ (1,1-diphenyl-2-picrylhydrazyl), ABTS^•+^ 2,2′-azinobis-(3-ethylbenzothiazoline-6-sulfonic acid), FRAP (Ferric Reducing Ability of Plasma) discoloration methods.

The total phenolic compounds (TPC) content of the algae extracts and the influence of two pre-treatments, solid-state fermentation with the *Lactiplantibacillus plantarum* LUHS135 and ultrasonication is given in Table 3. In comparison, the TPC content multivariate analysis of variance showed that algae species (*p* ≤ 0.0001), algae × pre-treatment before extraction interaction (*p* ≤ 0.0001) and algae species × LUHS135 combination interaction (*p* ≤ 0.003) had significant effects on TPC content in samples. The lowest TPC content in the non-pre-treated samples group was found in ClaR_non_, Ul_non_ and Sp_non_ samples (on average 1.18 mg GAE/mL), and the highest was found in ClaR_nonLUHS135_ and Furc_nonLUHS135_ samples (on average 13.28 mg GAE/mL). In comparison, for extracts and extracts × LUHS135 combinations prepared from ultrasonicated algae, the lowest TPC content was found in Sp_ultr_ samples (0.51 mg GAE/mL), and the highest TPC content was in ClaR_ultrLUHS135_, ClaG_ultrLUHS135_ and Furc_ultrLUHS135_ samples (on average 12.23 mg GAE/mL). Similar tendencies were established in the fermented samples group, and the lowest TPC content was found in Sp_ferm_ samples (2.77 mg GAE/mL) while the highest was in ClaR_fermLUHS135_ and Furc_fermLUHS135_ samples (on average 12.76 mg GAE/mL).

**Table 3:** Antioxidant activities and total phenolic compound content of algae extracts and algae extracts × LUHS135 combinations.

**Extracts and extract × LUHS135 combination**	**DPPH^•^, μmol TE/mL**	**ABTS^•+^, μmol TE/mL**	**FRAP, μmol TE/mL**	**TPC, mg GAE/mL**
Extracts and extracts × LUHS135 combinations prepared from non-pre-treated algae				
ClaR_non_	0.180 ± 0.017^a^	0.704 ± 0.032^a^	0.077 ± 0.006^b^	1.30 ± 0.095^a^
ClaR_nonLUHS135_	1.87 ± 0.141^f^	4.60 ± 0.092^f, g^	2.19 ± 0.210^h^	12.8 ± 0.032^f^
ClaG_non_	0.245 ± 0.028^b^	2.70 ± 0.071^d^	0.360 ± 0.034^c^	5.50 ± 0.158^b^
ClaG_nonLUHS135_	0.676 ± 0.046^c^	4.44 ± 0.110^f^	0.728 ± 0.063^e^	11.7 ± 0.140^e^
Furc_non_	1.52 ± 0.104^e^	3.68 ± 0.101^e^	0.869 ± 0.047^f^	9.76 ± 0.086^c^
Furc_nonLUHS135_	1.84 ± 0.093^f^	4.65 ± 0.152^f, g^	2.37 ± 0.235^h^	13.77 ± 0.160^f^
Ul_non_	0.197 ± 0.013^a^	2.20 ± 0.076^b^	0.063 ± 0.005^a^	1.15 ± 0.073^a^
Ul_nonLUHS135_	0.834 ± 0.079^d^	4.26 ± 0.095^f^	1.21 ± 0.114^g^	11.27 ± 0.079^d, e^
Sp_non_	0.187 ± 0.017^a^	2.44 ± 0.084^c^	0.051 ± 0.004^a^	1.10 ± 0.081^a^
Sp_nonLUHS135_	0.661 ± 0.056^c^	4.41 ± 0.141^f^	0.603 ± 0.037^d^	10.83 ± 0.011^d^
Extracts and extracts × LUHS135 combinations prepared from ultrasonicated algae				
ClaR_ultr_	0.288 ± 0.037^b^	2.37 ± 0.110^d^	1.14 ± 0.072^f^	6.38 ± 0.284^d^
ClaR_ultrLUHS135_	1.09 ± 0.093^f^	4.45 ± 0.312^g^	0.932 ± 0.064^e^	12.26 ± 0.546^f^
ClaG_ultr_	0.259 ± 0.035^b^	1.55 ± 0.091^c^	0.117 ± 0.009^c^	5.06 ± 0.216^c^
ClaG_ultrLUHS135_	1.02 ± 0.104^f^	4.52 ± 0.234^g^	0.540 ± 0.047^d^	12.19 ± 0.631^f^
Furc_ultr_	0.704 ± 0.078^d^	2.27 ± 0.155^d^	1.03 ± 0.084^f^	6.13 ± 0.277^d^
Furc_ultrLUHS135_	1.26 ± 0.088^f^	4.67 ± 0.191^g^	1.68 ± 0.086^g^	12.23 ± 0.495^f^
Ul_ultr_	0.403 ± 0.039^c^	1.33 ± 0.084^b^	0.058 ± 0.006^b^	1.85 ± 0.115^b^
Ul_ultrLUHS135_	0.762 ± 0.066^d^	4.26 ± 0.255^f^	1.23 ± 0.121^f^	11.16 ± 0.558^e^
Sp_ultr_	0.078 ± 0.010^a^	0.223 ± 0.027^a^	0.031 ± 0.013^a^	0.51 ± 0.045^a^
Sp_ultrLUHS135_	0.877 ± 0.049^e^	3.91 ± 0.214^e^	1.34 ± 0.114^f^	11.11 ± 0.533^e^
Extracts and extracts × LUHS135 combinations prepared from fermented algae				
ClaR^ferm^	0.288 ± 0.029^c^	3.42 ± 0.212^c^	0.274 ± 0.026^b^	7.07 ± 0.234^c^
ClaR^fermLUHS135^	1.63 ± 0.052^f^	5.04 ± 0.321^f^	1.82 ± 0.154^f^	12.70 ± 0.540 ^f^
ClaG^ferm^	0.202 ± 0.025^b^	2.21 ± 0.044^b^	0.227 ± 0.021^b^	7.06 ± 0.304c
ClaG^fermLUHS135^	0.819 ± 0.078^d^	4.43 ± 0.103^e^	1.10 ± 0.112^c^	11.43 ± 0.482^e^
Furc^ferm^	1.11 ± 0.130^e^	3.86 ± 0.094^d^	1.28 ± 0.123^c^	9.53 ± 0.270^d^
Furc^fermLUHS135^	1.45 ± 0.132^f^	5.36 ± 0.332^f^	1.68 ± 0.142^e^	12.81 ± 0.499^f^
Ul^ferm^	0.202 ± 0.012^b^	2.11 ± 0.073^b^	0.258 ± 0.027^b^	3.60 ± 0.245^b^
Ul^fermLUHS135^	0.891 ± 0.055^d^	4.52 ± 0.081^e^	1.17 ± 0.140^d^	11.24 ± 0.334^e^
Sp^ferm^	0.140 ± 0.008^a^	1.29 ± 0.050^a^	0.054 ± 0.006^a^	2.77 ± 0.142^a^
Sp^fermLUHS135^	1.12 ± 0.111^e^	4.79 ± 0.131^e^	1.34 ± 0.121^d^	11.75 ± 0.422^e^

ClaR, Cladophora rupestris; ClaG, Cladophora glomerata; Ul, Ulva intestinalis; Furc, Furcellaria lumbricalis; Sp, Spirulina (Arthrospira platensis); non, extracts prepared from non-pre-treated algae; ultr, extracts prepared from ultrasonicated algae; ferm, extracts prepared from fermented algae; LUHS135, extract × LUHS135 strain combination; DPPH^•^, 1,1-diphenyl-2-picrylhydrazyl; ABTS^•+^, 2,2′-azinobis-(3-ethylbenzothiazoline-6-sulfonic acid); FRAP, Ferric Reducing Ability of Plasma; TPC, total phenolic compounds content; GAE, gallic acid equivalents TE, Trolox equivalent; data are represented as means (n = 3 replicates of analysis) ± SE. a–h indicate the same analytical parameters for different algae species groups, and means with different letters are significantly different (p ≤ 0.05).

The antioxidant properties of two pre-treatments, solid-state fermentation with the *Lactiplantibacillus plantarum* LUHS135 and ultrasonication, on of macro- (*Cladophora rupestris, Cladophora glomerata, Furcellaria lumbricalis, Ulva intestinalis*) and Spirulina (*Arthrospira platensis*) extracts were estimated and compared by DPPH^•^, ABTS^•+^, and FRAP methods. In a comparison of the 2,2-diphenyl-1-picryhydrazyl (DPPH^•^) radical scavenging activity of all three groups of samples (non-pre-treated, ultrasonicated and fermented), multivariate analysis of variance showed that all of the analyzed factors and their interactions had significant effect on the DPPH^•^ radical scavenging activity of the samples (factors: algae species and pre-treatment before extraction (fermentation and/or ultrasonication), LUHS135 combination, algae species × LUHS135 combination interaction, algae extract × pre-treatment before extraction interaction, pre-treatment before extraction × LUHS135 combination interaction and the algae species × LUHS135 combination × pre-treatment before extraction interaction, *p* ≤ 0.0001). In comparison to the non-pre-treated (before extraction) samples group, the lowest DPPH^•^ radical scavenging activity was found in ClaR_non_, Ul_non_ and Sp_non_ samples (on average, 0.188 μmol TE/mL), and the highest DPPH^•^ radical scavenging activity was shown in ClaR_nonLUHS135_ and Furc_nonLUHS135_ samples (on average 1.86 μmol TE/mL). In extracts and extracts × LUHS135 combinations prepared from ultrasonicated algae, the lowest DPPH^•^ radical scavenging activity was found in Sp_ultr_ (0.078 μmol TE/mL); however, ClaR_ultrLUHS135_, ClaG_ultrLUHS135_ and Furc_ultrLUHS135_ samples showed an average of 14.4 times higher DPPH^•^ radical scavenging activity. Similar to the ultrasonicated group samples, in fermented samples we found the lowest DPPH^•^ radical scavenging activity in Sp_ferm_ samples (0.140 μmol TE/mL) and the highest in ClaR_fermLUHS135_ and Furc_fermLUHS135_ samples (on average 1.54 μmol TE/mL). Also, DPPH^•^ radical scavenging activity showed a weak positive correlation with samples' a^*^ coordinates (r = 0.231, *p* = 0.028). The -a^*^ and -b^*^ coordinates are related to chlorophyll's (-a and -b) greenish lipid-soluble pigments and causes the typical coloration of green algae (52, 53). However, carotenoids with a higher number of conjugated double bonds show red color and possess antioxidant properties (54). Other colored algae compounds with antioxidant properties are astaxanthin (52, 55–59) and canthaxanthin (β,β-carotene-4,4′-dione), which belongs to xanthophylls, and is widely used as a feed additive as an antioxidant (60–64).

2, 2′-azino-bis ethylbenzthiazoline-6-sulfonic acid (ABTS^•+^) radical cation scavenging of the samples showed similar tendencies to DPPH^•^ and FRAP, and a multivariate analysis of variance showed that all of the analyzed factors and their interactions had significant effects on sample ABTS^•+^ (algae species *p* ≤ 0.0001, pre-treatment before extraction *p* ≤ 0.0001, LUHS135 combination *p* ≤ 0.0001, algae species × LUHS135 combination interaction *p* = 0.015, algae extract × pre-treatment before extraction interaction *p* ≤ 0.0001, pre-treatment before extraction × LUHS135 combination interaction *p* ≤ 0.0001, algae species × LUHS135 combination × pre-treatment before extraction interaction *p* ≤ 0.0001). In comparison, in the non-pre-treated before extraction sample group, the lowest ABTS^•+^ was in ClaR_non_ samples (0.704 μmol TE/mL) and the highest was in ClaR_nonLUHS135_ and Furc_nonLUHS135_ samples (on average 4.63 μmol TE/mL). The highest ABTS^•+^ in the ultrasonicated group was from ClaR_ultrLUHS135_, ClaG_ultrLUHS135_ and Furc_ultrLUHS135_ samples (on average 4.55 μmol TE/mL) and the lowest was from Sp_ultr_ samples (0.223 μmol TE/mL). Similar tendencies were found in the fermented samples group: the lowest ABTS^•+^ was from Sp_ferm_ samples (1.29 μmol TE/mL) and the highest was from ClaR_fermLUHS135_ and Furc_fermLUHS135_ (on average 5.20 μmol TE/mL). ABTS^•+^ showed a weak, positive correlation with samples' a^*^ coordinates (r = 0.303, *p* = 0.004).

The ferric reducing antioxidant power (FRAP), which shows the ability of an antioxidant in reducing Fe(III) into Fe(II), demonstrated that all of the analyzed factors and their interactions had significant effects on the FRAP of the samples (*p* ≤ 0.0001). In comparison to the group that was not pre-treated before extraction, the lowest FRAP was established in Ul_non_ and Sp_non_ samples (on average 0.057 μmol TE/mL) and the highest FRAP was found in ClaR_nonLUHS135_ and Furc_nonLUHS135_ samples (on average 2.28 μmol TE/mL). In comparison to the ultrasonicated sample group, the lowest FRAP was found in Sp_ultr_ samples (0.031 μmol TE/mL) and the highest in Furc_ultrLUHS135_ samples (1.68 μmol TE/mL). In the fermented samples group, the lowest FRAP was in Sp_ferm_ samples (0.054 μmol TE/mL) and the highest was in ClaR_fermLUHS135_ (1.82 μmol TE/mL). FRAP showed a moderate negative correlation with the b^*^ coordinates of samples (r = 0.509, *p* = 0.0001). Phycobilin pigments are found in cyanobacteria and in the chloroplasts of red algae (52, 65). Liutein has a strong antioxidant effect (66). The main colored compounds in microalgae are fucoxanthin, lutein and β-carotene, and they also are described as good antioxidants (58, 59, 67–69). Zeaxanthin is a xanthophyll family carotenoid (70) and possesses antioxidant properties as well (71–75).

In essence, the radical scavenging activities of DPPH^•^ and ABTS^•+^ are based on the ability of antioxidants to donate a hydrogen atom or an electron to stabilize radicals by converting them to the non-radical species (76, 77). Our results reflected the ability of all prepared ethanolic extracts to donate a hydrogen atom or electron to both radicals. In general, algal extracts rich in natural polyphenolics can function as antioxidants (76, 78).

In this study, several methods based on different principles were used to determine the *in vitro* antioxidant activity of algae extracts. Other studies have reported that the FRAP method should be used in combination with other methods because it cannot measure all antioxidants of complex compounds (79, 80). Antioxidant properties of food and/or feed are desirable characteristics because antioxidants reduce oxidation processes (81). Also, it has been reported that both scavenging and antioxidant activities are related to TPC content (82). We found that TPC content in samples showed a moderate positive correlation with samples' a^*^ coordinates (r = 0.592, *p* = 0.0001), a negative weak correlation with samples' pH (r = −0.294, *p* = 0.005) and a moderate positive correlation with samples' ABTS^•+^ (r = 0.300, *p* = 0.004) and FRAP (r = 0.247, *p* = 0.019). However, a correlation between the DPPH^•^ and TPC content was not found. It was previously reported that in ethanolic extracts the correlation between TPC content and total antioxidant capacity is high, but the correlation with FRAP assay is minimal, and the correlation between the total antioxidant capacity and TPC content is positive and very significant in ethanolic extracts, whereas it is negative in methanolic ones (83). However, in the free form, phenolic compounds have a better bio-accessibility because of released free aglycones and increased antioxidative activity (84, 85), and fermentation could decrease free phenolic compound content in samples because they may bind with other molecules present in the fermentable matrix, i.e., might be hydrolysed and/or be degraded by microbial enzymes (42, 84). According to Li et al. (86), LAB fermentation has a significant impact on the phenolic profile, as well as on antioxidant activity, because during the process, various phenolic acids could be excreted to the fermentable matrix (86). It was reported that *Furcellaria* extracts, in comparison with *Cladophora* and *Ulva sp*., had the highest antioxidant activity of all the macroalgae alcoholic extracts tested (87). It has also been shown that the ethanolic extract of green and red seaweeds exhibit a high scavenging activity and a higher DPPH^•^ of brown and green seaweeds in comparison with red (83, 88–90). The lower correlation between FRAP values and TPC content in extracts shows that the phenolic compounds are not involved in the antioxidant activity through this pathway, but there might be some effects involving other active compounds (83). The current study showed that the combinations of extracts and LUHS135 could improve antioxidant properties of the substrate.

### Antimicrobial characteristics of the algal extracts

**Figure 3:**
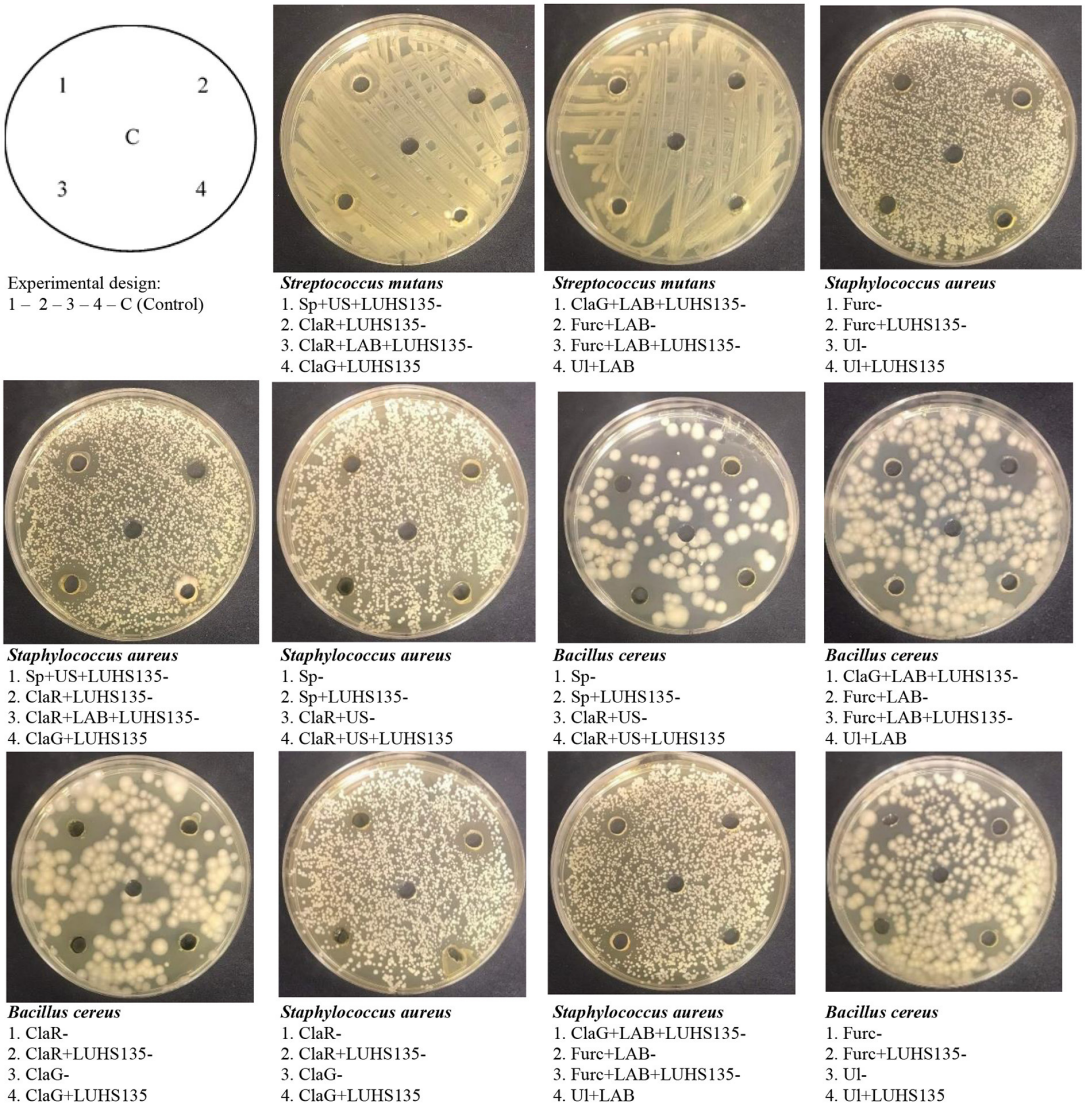
Images of the inhibition zones of the algae extracts and algae extracts × LUHS135 combinations evaluated using the agar well diffusion method [ClaR, Cladophora rupestris; ClaG, Cladophora glomerata; Ul, Ulva intestinalis; Furc, Furcellaria lumbricalis; Sp, Spirulina (Arthrospira platensis)]; US, algae biomass pre-treated with ultrasound; LAB, algae biomas fermented with LUHS135 strain before extraction; LUHS135, extract composition with LUHS135 strain; C,control (physiological solution)].

Antimicrobial activity of the algae extracts and algae extracts × LUHS135 combinations were evaluated using the agar well–diffusion method. The results are shown in Table 4 and Figure 3. In a comparison of all three groups (non-pre-treated, ultrasonicated and fermented), the highest number of samples (of all tested samples) that showed antimicrobial properties against at least one pathogen was found in the non-pre-treated samples group. All of the tested samples in this group showed inhibition properties against *Bacillus cereus* (the highest diameter of inhibition zones (DIZ), on average 16.0 mm, was found by ClaR_non_, ClaG_non_, ClaG_nonLUHS135_, Ul _nonLUHS135_ and Sp_nonLUHS135samples_). Also, 3 out of 10 samples of this group showed inhibition properties against *Enterococcus faecium* (ClaR_non_, ClaR_nonLUHS135_ and Cla_GnonLUHS135_, with DIZ of 15.3, 11.5 and 8.0 mm, respectively) and 4 out of 10 samples of this group showed inhibition properties against *Staphylococcus aureus* (ClaR_nonLUHS135_, ClaG_nonLUHS135_, Furc_nonLUHS135and_ Ul _nonLUHS135_, with DIZ of 12.4, 11.5, 12.3 and 8.0 mm, respectively). Despite the fact that the highest number of samples (of all tested samples) showed antimicrobial properties against at least one pathogen in the non-pre-treated samples group, a broader spectrum of pathogen inhibition was found in the ultrasonicated sample group (inhibition properties against *Bacillus cereus* showed in ClaR_ultr_, ClaR_ultrLUHS135_ and Sp_ultrLUHS135_ samples, inhibition properties against *Enterococcus faecium* showed in ClaG_ultrLUHS135_, inhibition properties against *Staphylococcus aureus* showed in ClaR_ultrLUHS135_, ClaG_ultrLUHS135_ and Sp_ultrLUHS135_ and inhibition properties against *Stretococcus mutans* showed in Furc_ultrLUHS135_ and Ul_ultrLUHS135_ samples). In the comparison of extract samples prepared from fermented algae, ClaR_ferm_, ClaR_fermLUHS135_ and Furc_fermLUHS135_ showed inhibition properties against one out of seven tested pathogens [ClaR_ferm_ and Furc_fermLUHS135_ inhibited *Bacillus cereus* (DIZ 16.3 and 13.1 mm, respectively) and Furc_fermLUHS135_ inhibited *Staphylococcus aureus* (DIZ 16.3 mm)]. Also, ClaG_fermLUHS135_ and Furc_ferm_ samples showed inhibition properties against both *Bacillus cereus* and *Staphylococcus aureus* strains (DIZ against *Bacillus cereus* 14.2 mm and 13.4 mm, respectively, and DIZ against *Staphylococcus aureus* 12.1 mm and 13.3 mm, respectively).

**Table 4:** Antimicrobial activity of the algae extracts and algae extracts × LUHS135 combinations evaluated using the agar well–diffusion method.

**Extracts and extract × LUHS135 combination**	**Pathogenic and opportunistic bacteria strain**						
	***Salmonella enterica***	***Bacillus cereus***	***Enterococcus faecium***	***Staphylococcus aureus***	***Escherichia coli***	***Streptococcus mutans***	***Enterococcus faecalis***
	**Diameter of the Inhibition zone, mm**						
**Extracts and extracts** **×LUHS135 combinations prepared from non-pre-treated algae**							
ClaR_non_	nd	16.1 ± 1.3^d^	15.3 ± 0.2^c^	nd	nd	nd	nd
ClaR_nonLUHS135_	nd	12.3 ± .2^b^	11.5 ± 0.2^b^	12.4 ± 0.4^c^	nd	nd	nd
ClaG_non_	nd	15.2 ± 0.6^d^	Nd	Nd	nd	nd	nd
ClaG_nonLUHS135_	nd	16.0 ± 0.3^d^	8.0 ± 0.1^a^	11.5 ± 0.3^b^	nd	nd	nd
Furc_non_	nd	13.4 ± 0.5^c^	nd	nd	nd	nd	nd
Furc_nonLUHS135_	nd	11.2 ± 0.1^a^	nd	12.3 ± 0.1^c^	nd	nd	nd
Ul_non_	nd	12.3 ± 0.3^b^	nd	Nd	nd	nd	nd
Ul _nonLUHS135_	nd	16.1 ± 0.2^d^	nd	8.0 ± 0.2^a^	nd	nd	nd
Sp_non_	nd	12.4 ± 0.2^b^	nd	Nd	nd	nd	nd
Sp_nonLUHS135_	nd	16.4 ± 0.3^d^	nd	Nd	nd	nd	nd
Extracts and extracts × LUHS135 combinations prepared from ultrasonicated algae							
ClaR_ultr_	nd	18.2 ± 0.5^b^	nd	Nd	nd	nd	nd
ClaR_ultrLUHS135_	nd	16.4 ± 0.2^a^	nd	14 ± 0.5^b^	nd	nd	nd
ClaG_ultr_	nd	nd	nd	nd	nd	nd	nd
ClaG_ultrLUHS135_	nd	nd	12.6 ± 0.4	8.0 ± 0.1^a^	nd	nd	nd
Furc_ultr_	nd	nd	nd	Nd	nd	nd	nd
Furc_ultrLUHS135_	nd	nd	nd	Nd	nd	8.0 ± 0.2^a^	nd
Ul_ultr_	nd	nd	nd	nd	nd	Nd	nd
Ul_ultrLUHS135_	nd	nd	nd	nd	nd	12 ± 0.3^b^	nd
Sp_ultr_	nd	nd	nd	nd	nd	Nd	nd
Sp_ultrLUHS135_	nd	18.1 ± 0.5^b^	nd	14.6 ± 0.6b	nd	Nd	nd
**Extracts and extracts** **×LUHS135 combinations prepared from fermented algae**							
ClaR_ferm_	nd	16.3 ± 0.6^c^	nd	Nd	nd	Nd	nd
ClaR_fermLUHS135_	nd	Nd	nd	15.4 ± 0.3^c^	nd	Nd	nd
ClaG_ferm_	nd	Nd	nd	nd	nd	Nd	nd
ClaG_fermLUHS135_	nd	14.2 ± 0.2^b^	nd	12.1 ± 0.1^a^	nd	Nd	nd
Furc_ferm_	nd	13.4 ± 0.4^a^	nd	13.3 ± 0.2^b^	nd	Nd	nd
Furc_fermLUHS135_	nd	13.1 ± 0.1^a^	nd	nd	nd	Nd	nd
Ul_ferm_	nd	nd	nd	nd	nd	Nd	nd
Ul_fermLUHS135_	nd	nd	nd	nd	nd	Nd	nd
Sp_ferm_	nd	nd	nd	nd	nd	Nd	nd
Sp_fermLUHS135_	nd	nd	nd	nd	nd	Nd	nd

ClaR, Cladophora rupestris; ClaG, Cladophora glomerata; Ul, Ulva intestinalis; Furc, Furcellaria lumbricalis; Sp, Spirulina (Arthrospira platensis); non, extracts prepared from non-pre-treated algae; ultr, extracts prepared from ultrasonicated algae; ferm, extracts prepared from fermented algae; LUHS135, extract × LUHS135 strain combination; nd, not determined; data are represented as means (n = 3 replicates of analysis) ± SE. a–d indicate the same analytical parameters in different algae species group, and means with different letters are significantly different (p ≤ 0.05).

The results of antimicrobial activity of the algae extracts and algae extracts × LUHS135 combinations evaluated in liquid medium by testing concentrations of algae extract and/or the algae extract × LUHS135 combination in 500 and 2000 μL concentrations and pathogen concentration of 10 μL are shown in Tables 5, 6, respectively. We found that at a concentration of 500 μL in liquid medium, Sp_non_ samples inhibited *Bacillus cereus* growth, ClaR_ferm_ samples inhibited *Enterococcus faecium* growth, Sp_ultrLUHS135_, ClaR_ferm_ and Sp_fermLUHS135_ samples inhibited *Staphylococcus aureus* growth and Sp_non_ samples inhibited *Streptococcus mutans* growth. By increasing algae extract and algae extracts × LUHS135 combinations concentrations to 2000 μL, in addition to the mentioned antimicrobial properties, *Enterococcus faecium* was also inhibited by ClaR_non_, Sp_non_, Sp_nonLUHS135_ and Ul_ultr_ samples, *Streptococcus mutans* was inhibited by ClaR_ultr_, Ul_ultr_ and Sp_ferm_ samples and *Enterococcus faecalis* was inhibited by ClaR_non_, ClaG_non_, Sp_non_ and ClaG_ferm_ samples.

**Table 5:** Antimicrobial activity of the algae extracts and algae extracts × LUHS135 combinations evaluated in liquid medium by testing concentration of algae extract and/or algae extract × LUHS135 combination at a concentration of 500 μL and pathogen concentration at 10 μL.

**Extracts and extract × LUHS135 combination**	**Pathogenic and opportunistic bacteria strains**						
	***Salmonella enterica***	***Bacillus cereus***	***Enterococcus faecium***	***Staphylococcus aureus***	***Escherichia coli***	***Streptococcus mutans***	***Enterococcus faecalis***
**Inhibition zone, mm**							
**Extracts and extracts** **×LUHS135 combinations prepared from non-pre-treated algae**							
**Concentration of algae extract 500** ***μ*****L, concentration of pathogen 10** ***μ*****L**							
ClaR_non_	+	+	+	+	+	+	+
ClaR_nonLUHS135_	+	+	+	+	+	+	+
ClaG_non_	+	+	+	+	+	+	+
ClaG_nonLUHS135_	+	+	+	+	+	+	+
Furc_non_	+	+	+	+	+	+	+
Furc_nonLUHS135_	+	+	+	+	+	+	+
Ul_non_	+	+	+	+	+	+	+
Ul _nonLUHS135_	+	+	+	+	+	+	+
Sp_non_	+	-	+	+	+	-	+
Sp_nonLUHS135_	+	+	+	+	+	+	+
**Extracts and extracts** **×LUHS135 combinations prepared from ultrasonicated algae**							
**Concentration of algae extract 500** ***μ*****L, concentration of pathogen 10** ***μ*****L**							
ClaR_ultr_	+	+	+	+	+	+	+
ClaR_ultrLUHS135_	+	+	+	+	+	+	+
ClaG_ultr_	+	+	+	+	+	+	+
ClaG_ultrLUHS135_	+	+	+	+	+	+	+
Furc_ultr_	+	+	+	+	+	+	+
Furc_ultrLUHS135_	+	+	+	+	+	+	+
Ul_ultr_	+	+	+	+	+	+	+
Ul_ultrLUHS135_	+	+	+	+	+	+	+
Sp_ultr_	+	+	+	+	+	+	+
Sp_ultrLUHS135_	+	+	+	-	+	+	+
**Extracts and extracts** **×LUHS135 combinations prepared from fermented algae**							
**Concentration of algae extract 500** ***μ*****L, concentration of pathogen 10** ***μ*****L**							
ClaR_ferm_	+	+	-	-	+	+	+
ClaR_fermLUHS135_	+	+	+	+	+	+	+
ClaG_ferm_	+	+	+	+	+	+	+
ClaG_fermLUHS135_	+	+	+	+	+	+	+
Furc_ferm_	+	+	+	+	+	+	+
Furc_fermLUHS135_	+	+	+	+	+	+	+
Ul_ferm_	+	+	+	+	+	+	+
Ul_fermLUHS135_	+	+	+	+	+	+	+
Sp_ferm_	+	+	+	+	+	+	+
Sp_fermLUHS135_	+	+	+	-	+	+	+
**Pathogen control**							
Pathogen	+	+	+	+	+	+	+

Interpretation of results: negative (–) means the pathogens did not grow on the selective culture medium; positive (+) means the pathogens grew on the selective culture medium; ClaR, Cladophora rupestris; ClaG, Cladophora glomerata; Ul, Ulva intestinalis; Furc, Furcellaria lumbricalis; Sp, Spirulina (Arthrospira platensis); non, extracts prepared from non-pre-treated algae; ultr, extracts prepared from ultrasonicated algae; ferm, extracts prepared from fermented algae; LUHS135, extract × LUHS135 strain combination.

**Table 6:** Antimicrobial activity of the algae extracts and algae extracts × LUHS135 combinations evaluated in liquid medium by testing the concentration of algae extract and/or algae extract × LUHS135 combination at a concentration of 2,000 μL and pathogen concentration at 10 μL.

**Extracts and extract × LUHS135 combination**	**Pathogenic and opportunistic bacteria strains**						
	***Salmonella enterica***	***Bacillus cereus***	***Enterococcus faecium***	***Staphylococcus aureus***	***Escherichia coli***	***Streptococcus mutans***	***Enterococcus faecalis***
**Inhibition zone, mm**							
**Extracts and extracts** **×LUHS135 combinations prepared from non-pre-treated algae**							
**Concentration of algae extract 2,000** ***μ*****L, concentration of pathogen 10** ***μ*****L**							
ClaR_non_	+	+	-	+	+	+	-
ClaR_nonLUHS135_	+	+	+	+	+	+	+
ClaG_non_	+	+	+	+	+	+	-
ClaG_nonLUHS135_	+	+	+	+	+	+	+
Furc_non_	+	+	+	+	+	+	+
Furc_nonLUHS135_	+	+	+	+	+	+	+
Ul_non_	+	+	-	+	+	+	+
Ul _nonLUHS135_	+	+	+	+	+	+	+
Sp_non_	+	-	-	+	+	-	-
Sp_nonLUHS135_	+	+	-	+	+	+	+
**Concentration of algae extract 2,000** ***μ*****L, concentration of pathogen 10** ***μ*****L**							
ClaR_ultr_	+	+	+	+	+	-	+
ClaR_ultrLUHS135_	+	+	+	+	+	+	+
ClaG_ultr_	+	+	+	+	+	+	+
ClaG_ultrLUHS135_	+	+	+	+	+	+	+
Furc_ultr_	+	+	+	+	+	+	+
Furc_ultrLUHS135_	+	+	+	+	+	+	+
Ul_ultr_	+	+	-	+	+	-	+
Ul_ultrLUHS135_	+	+	+	+	+	+	+
Sp_ultr_	+	+	+	+	+	+	+
Sp_ultrLUHS135_	+	+	+	-	+	+	+
**Extracts and extracts** **×LUHS135 combinations prepared from fermented algae**							
**Concentration of algae extract 2,000** ***μ*****L, concentration of pathogen 10** ***μ*****L**							
ClaR_ferm_	+	+	-	-	+	+	+
ClaR_fermLUHS135_	+	+	+	+	+	+	+
ClaG_ferm_	+	+	+	+	+	+	-
ClaG_fermLUHS135_	+	+	+	+	+	+	+
Furc_ferm_	+	+	+	+	+	+	+
Furc_fermLUHS135_	+	+	+	+	+	+	+
Ul_ferm_	+	+	+	+	+	+	+
Ul_fermLUHS135_	+	+	+	+	+	+	+
Sp_ferm_	+	+	+	+	+	-	+
Sp_fermLUHS135_	+	+	+	-	+	+	+
**Pathogen control**							
Pathogen	+	+	+	+	+	+	+

Interpretation of results: negative (–) means the pathogens did not grow on the selective culture medium; positive (+) means the pathogens grew on the selective culture medium; ClaR, Cladophora rupestris; ClaG, Cladophora glomerata; Ul, Ulva intestinalis; Furc, Furcellaria lumbricalis; Sp, Spirulina (Arthrospira platensis); non, extracts prepared from non-pre-treated algae; ultr, extracts prepared from ultrasonicated algae; ferm, extracts prepared from fermented algae; LUHS135, extract × LUHS135 strain combination.

Algae are a good source of bioactive compounds, and some of them possess broad spectrum activities, including antimicrobial activities (3, 91, 92). *Bacillus cereus* is a facultative aerobic spore-forming bacterium (93, 94), and is a well-known foodborne pathogen that is able to grow in the intestinal tracts of insects and mammals (94). *Ulva* species inhibit the growth of some Gram-positive pathogens (*Bacillus cereus* and *Staphylococcus aureus*) at ≤ 500 μg/mL concentration (95). Gram-positive bacteria are more susceptible to algae extracts, in comparison with Gram-negative bacteria, which is explained by extracts' compositions (high concentration of phenolic compounds, terpenoids, alkaloids, etc.), which damage the cellular wall. In contrast, the external membrane of Gram-negative bacteria acts as a barrier, preventing any substance from passing through (96). Among the predominant human pathogens, *Staphylococcus aureus* is the foremost cause of gastroenteritis (94, 97). *Cladophora rupestris* inhibits *S. aureus* growth (with DIZ 16.3 mm) (98, 99). Also, ethanolic extracts of *Cladophora* sp. possess stronger antibacterial activity against *S. aureus* in comparison with *Ulva sp*. extracts (96). However, different compositions of extrahent can lead to different properties of the extracts (96). In red seaweeds, including *F. lumbricalis*, strong inhibition properties against *S. aureus* were also reported (99–102), and it is thought that red types of seaweed are very promising agents against *S. aureus* (99). Also, Gram-positive bacteria (*B. cereus* and *S. aureus*) showed higher sensitivity to Spirulina extracts in comparison with Gram-negative ones (103). Elshouny et al. (104) reported, that Spirulina possesses antimicrobial activity against not only *S. aureus*, but also inhibits *E. coli* and *Salmonella* spp. growth. Mohammed et al. (105) reported, that Gram-positive strains are more sensitive to *Cladophora, Spirulina platensis* and *S. glomerata* extracts than Gram-negative ones, and the highest inhibitory efficacy was found to be against *S. aureus* (105). Another pathogenic and opportunistic strain, *E. faecium*, is a significant opportunistic human pathogen with a broad host range (106). *Enterococcus faecium* causes big problems because of its broad resistance to antimicrobials (106). From this point of view, natural antimicrobials, which could be used for opportunistic pathogenic strain inhibition, become very important. *Streptococcus mutans* can cause dental decay (107, 108), and some *S. mutans* proteins contribute to the pathogenesis of *S. mutans* by promoting adherence to dental plaque (107, 109–112). Also, Sirbu et al. (113) reported that TPC in algae extracts is related with their antibacterial activity. In this study we established that there are moderate correlations between ABTS^•+^ and *E. faecalis* DIZ and between the TPC content in extracts and *S. aureus* DIZ (r = 0.388, *p* = 0.0001; r = 0.340, *p* = 0.001, respectively). However, further research is needed to evaluate which compounds are responsible for the inhibition of these pathogens.

## Conclusions

This study confirmed, that the species of algae is significant factor on samples pH (*p* = 0.017) and 2% of yeast extract leads to more effective fermentation of algal biomass, as after 36 h of SSF, significant lower algae pH values were obtained. The highest DPPH^•^, ABTS^•+^, and FRAP antioxidant properties were shown by non-pretreated *Cladophora rupestris* and *Furcellaria lumbricalis* extract combinations with LUHS135, in comparison with extracts without LUHS135. A moderate positive correlation of TPC with samples ABTS^•+^ (r = 0.300, *p* = 0.004) and FRAP (r = 0.247, *p* = 0.019) was established, however, between samples DPPH^•^ and TPC content correlations were not found. Despite, that in the non-pre-treated samples group the highest number of samples showed antimicrobial properties at least against one pathogen, a broader spectrum of pathogens inhibition showed ultrasonicated samples group (inhibited 4 out of 7 tested pathogens). Finally, despite, that the extract combinations with LUHS135 strain showed prospective results, further research is needed to evaluate, which compounds are responsible for antioxidant properties of the extracts, as well as pathogens inhibition.

## Data availability statement

The raw data supporting the conclusions of this article will be made available by the authors, without undue reservation.

## Author contributions

Conceptualization: EB, PV, and MR. Methodology: EB, PV, DU, and MR. Software, validation, writing—original draft, and preparation: ET and EB. Formal analysis: VS, EZ, DU, and RR. Investigation: EB, ET, and MR. Resources, supervision, and project administration: EB. Data curation: ET. Writing—review and editing: EB, PV, MR, RP, and JR. Visualization: ET, VS, and EZ. All authors have read and agreed to the published version of the manuscript.

## Conflict of interest

The authors declare that the research was conducted in the absence of any commercial or financial relationships that could be construed as a potential conflict of interest.

## Publisher's note

All claims expressed in this article are solely those of the authors and do not necessarily represent those of their affiliated organizations, or those of the publisher, the editors and the reviewers. Any product that may be evaluated in this article, or claim that may be made by its manufacturer, is not guaranteed or endorsed by the publisher.
